# Supplementary material for: Foam-in-Vein: Characterisation of Blood Displacement Efficacy of Liquid Sclerosing Foams
Source: Biomolecules. 2022 Nov 22;12(12):1725. doi: 10.3390/biom12121725 (PMC9775758; doi:10.3390/biom12121725)
Supplement: Supplementary file 1 [file biomolecules-12-01725-s001.zip › biomolecules-1858514-supplementary.pdf]

Supplementary Material for:

Foam-in-Vein: Characterisation of Blood Displacement Efficacy of Liquid Sclerosing Foams

S.1 Pipe Viscometry

Pressure signals were recorded as .csv files at 10 Hz acquisition frequency *via* the Elveflow Software Interface (ESI) supplied by the sensor manufacturer. Raw data were processed with Python scripts written to detect the plateau region and calculate pressure differentials, and to plot the resulting rheograms. To minimise instrument noise and smooth the data, a Savitzky-Golay filter was applied, with a window length of 15 and a polyorder of 2. In order to identify the initial and final points of the pressure plateau region (P1 and P2), the "ginput" function was called in Python to define a local region which was then used by the script to identify P1 (local maximum) and P2 (local first differential minimum). **Figure S1** shows a representative example of the plateau detection process. The plateau region was subsequently averaged for each repeat (ΔP_{ave}) and the corresponding standard deviation was calculated. ΔP_{ave} of three repeats were averaged to result in a mean pressure change ($\overline{\Delta P}$) which was then used to calculate wall shear stress (τ_w).

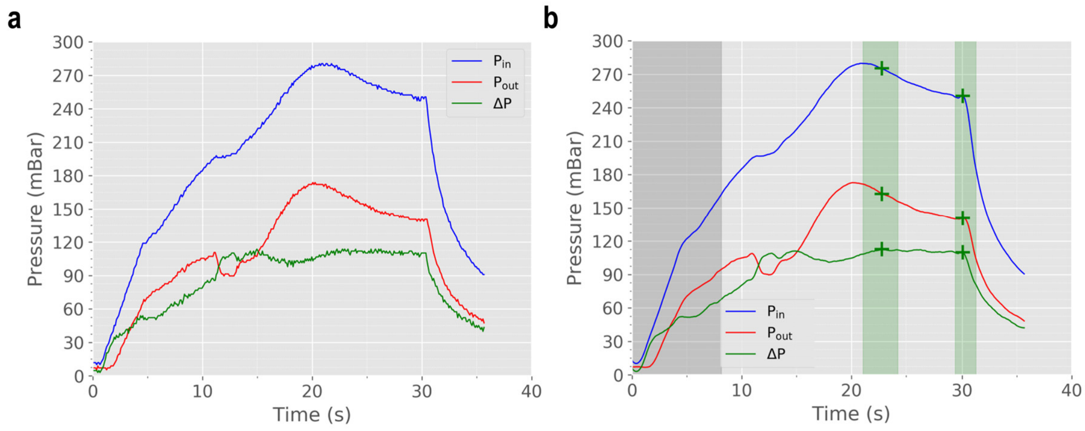

Figure S1. Processing of pressure data. (a) Raw data obtained from the ESI software. (b) Processed data, showing the loci of first and final points of the ΔP plateau region (grey region: the time it takes for the tube to be fully primed with foam). Data were first smoothened using a Savitzky – Golay filter (window length = 15, polyorder = 2). The green regions highlight the user-selected local areas that the Python algorithm uses to compute P1 and P2 (marked as **+**). Image taken from [1].

The apparatus shown in **Figure S2**a was employed to conduct the pipe viscometry experiments. In order to securely connect the pressure sensors to the PTFE tube, two conical shape connectors (**Figure S2**b) were manufactured using polydimethylsiloxane (PDMS) polymer, from a mixture of monomer and curing agent (Sylgard® 184, Dow Corning Corporation, USA) at a 10:1 w/w ratio. The mixture was poured onto a PLA mould with a PVA core (**Figure S3**), degassed and cured at room temperature for 72 hours. The cured PDMS block was then placed in a water bath at 50 °C for seven days to dissolve the PVA core.

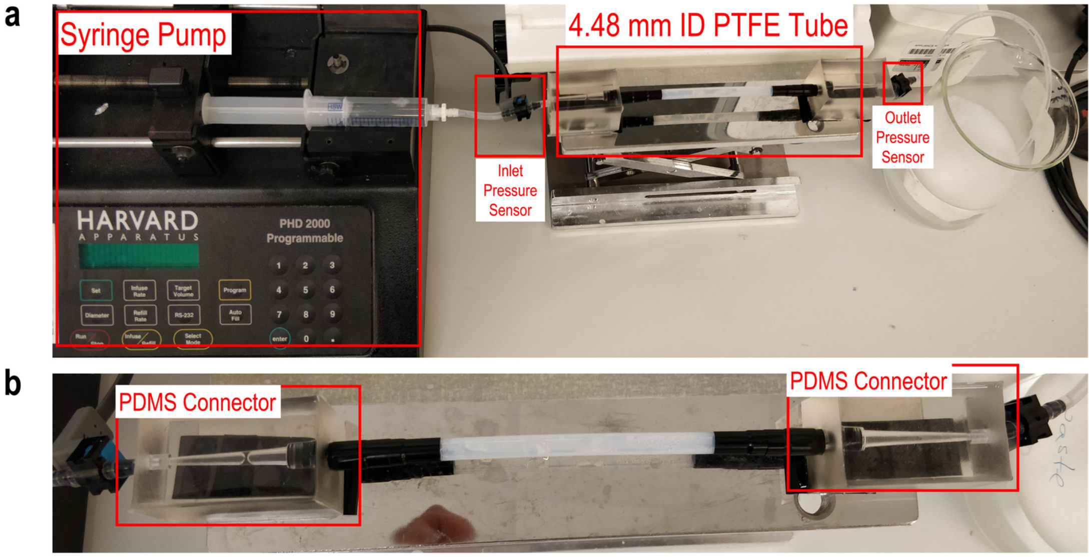

Figure S2. The pipe viscometry apparatus. (a) The 4.48 mm ID PTFE tube of length 15 cm used for the pipe viscometry experiment. The pressure sensors are connected to the PDMS connectors *via* silicone tubes that fit securely inside the PDMS blocks. (b) Top view of measurement region of the 4.48 mm PTFE tube. The PDMS connectors allowed for a smooth transition of diameter (note that the experiments were both conducted using the Harvard Apparatus syringe pump and this figure is only for demonstration purposes). Image adapted from [1].

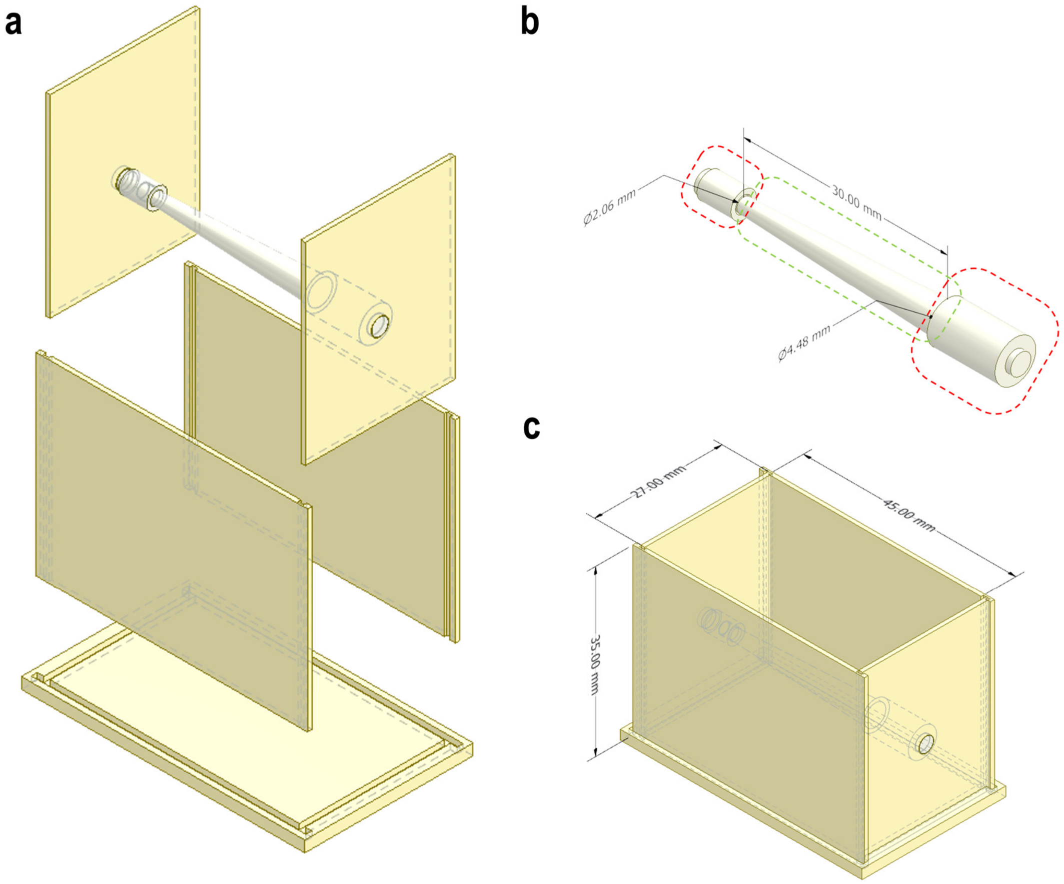

Figure S3. Connector mould assembly. PLA is labelled in yellow, while PVA is labelled in white. (a) The 3D-printed pieces are fitted together by sliding the pieces in place. (b) The water soluble PVA piece. The diameter transition region is 30 mm in length and has a diameter of 2.06 mm on one side (corresponding to the ID of the silicone tubes connected to the pressure transducers) and a diameter of 4.48 mm on the other side (corresponding to the ID of the PTFE tube). (c) Corner view of the assembled mould illustrating the dimensions of the PDMS block. Image taken from [1].

S.2 Rheograms of Carboxymethyl Cellulose and Blood

Figure S4 shows a comparison between viscosity profiles of blood [2] and various CMC solutions [3] calculated using the Cross model. The 0.2% CMC solution exhibits the closest rheological behaviour to blood under experimental and venous shear rates, and is thus the most appropriate blood substitute for the blood displacement experiments in this study.

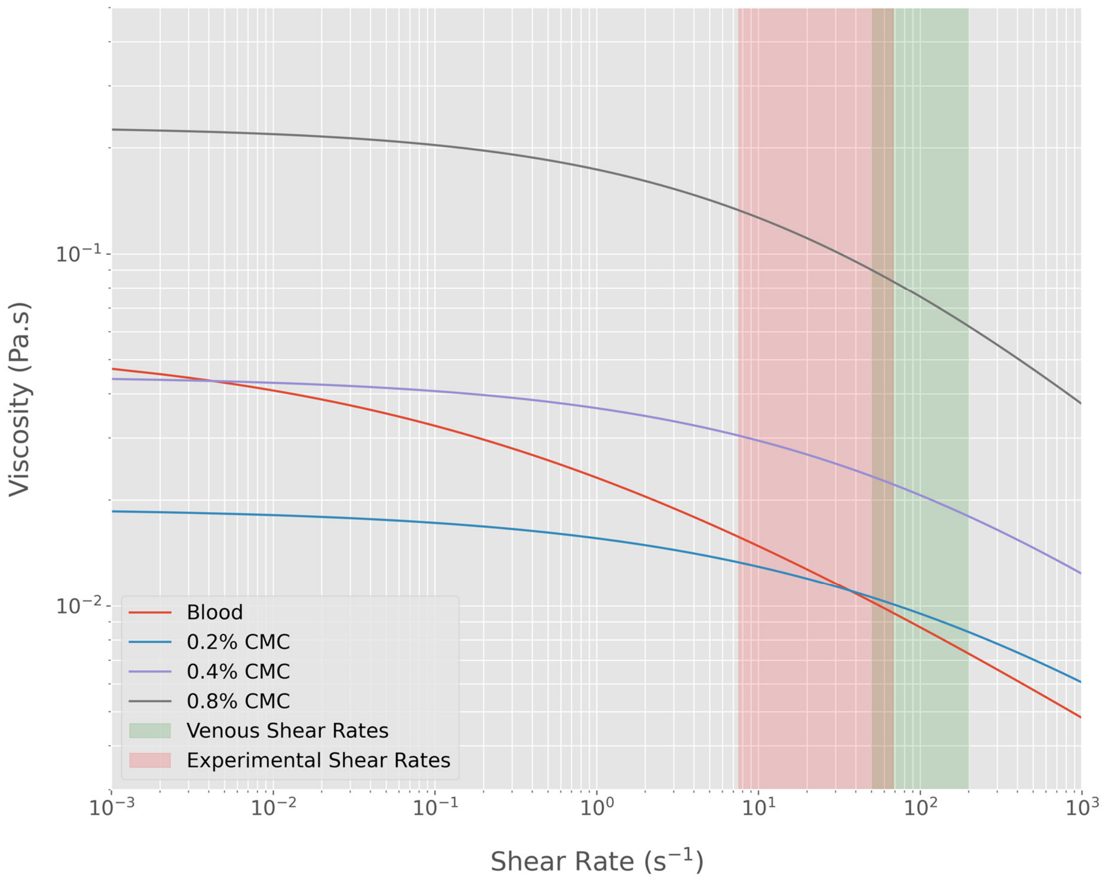

Figure S4. Comparison of viscosity profiles between human whole blood and CMC solutions (concentrations as % w/v) using the Cross model. Rheology of blood falls between 0.2% and 0.4% CMC solutions. Under venous shear rates and shear rates experienced in the 4.48 mm PTFE tube under experimental flowrates herein, the 0.2% CMC solution mimics the rheology of blood more closely, making it the best candidate for rheological replication of blood.

S.3 Determination of Power-law Indices

Using Prism 9, the slopes (n) and y-intercepts ($\ln K$) were calculated alongside their associated standard error of mean (SEM).

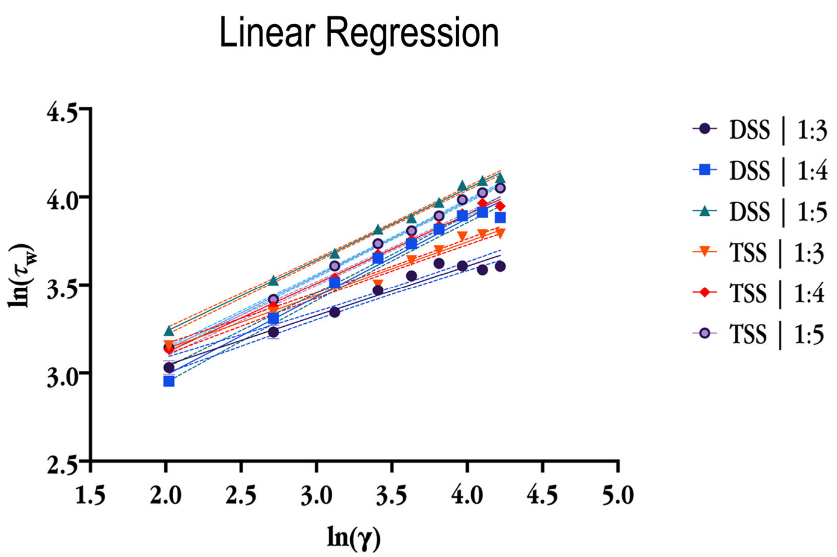

Figure S5. Linear regression of the linearised power law model ($\ln(\tau_w) = n.\ln \dot{\gamma}_{obs} + \ln K$) using Prism 9. Data were obtained *via* pipe viscometry of sclerosing foams of different L:G ratios (1:3, 1:4 and 1:5) produced *via* different formulation techniques (DSS and Tessari) injected into a PTFE tube of 4.48 mm ID. Dotted lines signify a 95% confidence interval due to the error of regression. Image taken from [1].

The power-law indices were thus derived and are reported in **Table S1**.

Table S1. Power-law indices obtained through linear regression analysis of $\ln \tau_w$ versus $\ln \dot{\gamma}_{obs}$ data using Prism. Associated SEMs were automatically calculated by Prism.

#	Tube ID	Formulation	L:G	n	SEM(n)	K	SEM(K)
1	4.48	DSS	1:3	0.2819	0.01369	11.93	0.5732
2			1:4	0.4453	0.01276	8.142	0.3647
3			1:5	0.4062	0.00597	11.27	0.2362
4		TSS	1:3	0.3051	0.00941	12.49	0.4126
5			1:4	0.3929	0.00647	10.27	0.2331
6			1:5	0.4237	0.00482	9.786	0.1655

S.4 Asymptotic and Linear Regression Results

Table S2. Asymptotic regression results of CMC displacement data. V_0 and κ are the asymptotic parameters while σ_{V_0} and σ_κ are their respective standard deviations.

$L:G$ ratio	V_0	σ_{V_0}	κ	σ_κ
**1:3**	5.63775435	0.10819937	1.15149322	0.09884661
**1:4**	5.41854763	0.06331562	0.92289887	0.03945151
**1:5**	5.40237701	0.05415715	0.70248649	0.02746466

Table S3. Asymptotic regression results of CMC displacement data per unit volume of foam-constituent gas. V_0/V_g and κ are the asymptotic parameters while σ_{V_0/V_g} and σ_κ are their respective standard deviations.

$L:G$ ratio	V_0/V_g	σ_{V_0/V_G}	κ	σ_κ
**1:3**	1.409439027	0.027050833	1.151491546	0.098848849
**1:4**	1.083709508	0.012663148	0.922898935	0.039451556
**1:5**	0.900395971	0.009026316	0.702487116	0.027464879

S.5 Results of 2-Way ANOVA on Gas-Normalised Displacement Data

Table S4. Statistical analysis of gas-normalised CMC displacement data obtained *via* 2-way ANOVA in Prism 9.

**Comparison of Flowrates**				
**Flowrate (mL.min^{-1})**	Formulation Technique	L:G ratio	Adjusted p-value	Summary
**4 vs 14**	DSS	1:3	<0.001	***
**4 vs 24**	DSS	1:3	<0.001	***
**4 vs 36**	DSS	1:3	<0.001	***
**14 vs 24**	DSS	1:3	<0.001	***
**14 vs 36**	DSS	1:3	<0.001	***
**24 vs 36**	DSS	1:3	<0.001	***
**4 vs 14**	DSS	1:4	<0.001	***
**4 vs 24**	DSS	1:4	<0.001	***
**4 vs 36**	DSS	1:4	<0.001	***
**14 vs 24**	DSS	1:4	<0.001	***
**14 vs 36**	DSS	1:4	<0.001	***
**24 vs 36**	DSS	1:4	<0.001	***
**4 vs 14**	DSS	1:5	<0.001	***
**4 vs 24**	DSS	1:5	<0.001	***
**4 vs 36**	DSS	1:5	<0.001	***
**14 vs 24**	DSS	1:5	<0.001	***

**14 vs 36**	DSS	1:5	<0.001	***
**24 vs 36**	DSS	1:5	<0.001	***
**4 vs 14**	TSS	1:3	<0.001	***
**4 vs 24**	TSS	1:3	<0.001	***
**4 vs 36**	TSS	1:3	<0.001	***
**14 vs 24**	TSS	1:3	<0.001	***
**14 vs 36**	TSS	1:3	<0.001	***
**24 vs 36**	TSS	1:3	<0.001	***
**4 vs 14**	TSS	1:4	<0.001	***
**4 vs 24**	TSS	1:4	<0.001	***
**4 vs 36**	TSS	1:4	<0.001	***
**14 vs 24**	TSS	1:4	<0.001	***
**14 vs 36**	TSS	1:4	<0.001	***
**24 vs 36**	TSS	1:4	<0.001	***
**4 vs 14**	TSS	1:5	<0.001	***
**4 vs 24**	TSS	1:5	<0.001	***
**4 vs 36**	TSS	1:5	<0.001	***
**14 vs 24**	TSS	1:5	<0.001	***
**14 vs 36**	TSS	1:5	<0.001	***
**24 vs 36**	TSS	1:5	<0.001	***

Comparison of Formulation Techniques

Flowrate (mL.min^{-1})	**Formulation Technique**	L:G ratio	Adjusted p-value	Summary
4	**DSS vs TSS**	1:3	0.7760	ns
4	**DSS vs TSS**	1:4	0.5711	ns
4	**DSS vs TSS**	1:5	0.6034	ns
14	**DSS vs TSS**	1:3	<0.001	***
14	**DSS vs TSS**	1:4	<0.001	***
14	**DSS vs TSS**	1:5	0.9987	ns
24	**DSS vs TSS**	1:3	0.9977	ns
24	**DSS vs TSS**	1:4	0.8147	ns
24	**DSS vs TSS**	1:5	>0.9999	ns
36	**DSS vs TSS**	1:3	>0.9999	ns
36	**DSS vs TSS**	1:4	0.9989	ns
36	**DSS vs TSS**	1:5	0.2270	ns

Comparison of L:G ratio

Flowrate (mL.min^{-1})	Formulation Technique	**L:G ratio**	Adjusted p-value	Summary
4	DSS	**1:3 vs 1:4**	<0.001	***
4	DSS	**1:3 vs 1:5**	<0.001	***
4	DSS	**1:4 vs 1:5**	<0.001	***
4	TSS	**1:3 vs 1:4**	<0.001	***
4	TSS	**1:3 vs 1:5**	<0.001	***
4	TSS	**1:4 vs 1:5**	<0.001	***

14	DSS	**1:3 vs 1:4**	<0.001	***
14	DSS	**1:3 vs 1:5**	<0.001	***
14	DSS	**1:4 vs 1:5**	<0.001	***
14	TSS	**1:3 vs 1:4**	<0.001	***
14	TSS	**1:3 vs 1:5**	<0.001	***
14	TSS	**1:4 vs 1:5**	<0.001	***
24	DSS	**1:3 vs 1:4**	<0.001	***
24	DSS	**1:3 vs 1:5**	<0.001	***
24	DSS	**1:4 vs 1:5**	<0.001	***
24	TSS	**1:3 vs 1:4**	<0.001	***
24	TSS	**1:3 vs 1:5**	<0.001	***
36	TSS	**1:4 vs 1:5**	<0.001	***
36	DSS	**1:3 vs 1:4**	<0.001	***
36	DSS	**1:3 vs 1:5**	<0.001	***
36	DSS	**1:4 vs 1:5**	<0.001	***
36	TSS	**1:3 vs 1:4**	<0.001	***
36	TSS	**1:3 vs 1:5**	<0.001	***
36	TSS	**1:4 vs 1:5**	<0.001	***

References

1. Meghdadi, A., et al., *Foam-in-vein: Rheological characterisation of liquid sclerosing foams using a pipe viscometer.* Colloids and Surfaces A: Physicochemical and Engineering Aspects, 2022. **645**: p. 128916.
2. Shibeshi, S.S. and W.E. Collins, *The rheology of blood flow in a branced arterial system.* Applied Rheology, 2005. **15**(6): p. 398-405.
3. Benchabane, A. and K. Bekkour, *Rheological properties of carboxymethyl cellulose (CMC) solutions.* Colloid and Polymer Science, 2008. **286**(10): p. 1173-1180.
